# Mass Transfer Enhancement in High-Solids Anaerobic Digestion of Organic Fraction of Municipal Solid Wastes: A Review

**DOI:** 10.3390/bioengineering10091084

**Published:** 2023-09-14

**Authors:** Qingwei Gao, Lili Li, Kun Wang, Qingliang Zhao

**Affiliations:** State Key Laboratory of Urban Water Resources and Environment (SKLUWRE), School of Environment, Harbin Institute of Technology, Harbin 150090, China

**Keywords:** high-solids anaerobic digestion, municipal solid wastes, rheological characteristics, mass transfer

## Abstract

The increasing global population and urbanization have led to a pressing need for effective solutions to manage the organic fraction of municipal solid waste (OFMSW). High-solids anaerobic digestion (HS-AD) has garnered attention as a sustainable technology that offers reduced water demand and energy consumption, and an increased biogas production rate. However, challenges such as rheology complexities and slow mass transfer hinder its widespread application. To address these limitations, this review emphasizes the importance of process optimization and the mass transfer enhancement of HS-AD, and summarizes various strategies for enhancing mass transfer in the field of HS-AD for the OFMSW, including substrate pretreatments, mixing strategies, and the addition of biochar. Additionally, the incorporation of innovative reactor designs, substrate pretreatment, the use of advanced modeling and simulation techniques, and the novel conductive materials need to be investigated in future studies to promote a better coupling between mass transfer and methane production. This review provides support and guidance to promote HS-AD technology as a more viable solution for sustainable waste management and resource recovery.

## 1. Introduction

With the exponential population growth, economic development, and rapid urbanization, the increased generation of municipal solid wastes has become a pressing concern for waste management [1]. The inherent complexity and heterogeneity of organic solid wastes demand the fine-tuning of process design for optimal waste utilization and the minimization of environmental impact. High-solids anaerobic digestion (HS-AD) of the organic fraction of municipal solid waste (OFMSW) has garnered significant attention as a promising anaerobic digestion (AD) technology for sustainable waste treatment and resource recovery [2]. For HS-AD, the total solids (TS) concentration would reach ≥15% [3,4], and it exhibits advantages such as flexible feedstock types, low water demand, a small reactor volume, high organics loading, and less heat consumption compared to the traditional low-solids anaerobic digestion (LS-AD) [5,6]. Additionally, the digestate produced as a by-product of the HS-AD process serves as a nutrient-rich fertilizer, aligning with circular economy principles and promoting sustainable agricultural practices [7]. The bibliometric search was performed using the Web of Science database with search parameters of “high solids anaerobic digestion” or “dry anaerobic digestion”. At the end of this process, the total set of literature across all databases from the year 2000 until 2023 was 13,304 papers and 1413 patents. Most studies had been conducted on a lab scale, with only 558 studies at the pilot scale and 109 studies at the real scale. The main HS-AD topics covered include the transformation of multiple organic matter and operational performance, methane production, mass transfer, and pretreatment.

Despite its numerous advantages, HS-AD is always accompanied by difficult mass transfer due to a low moisture content, leading to longer degradation times of organic matter and the accumulation of toxic and inhibitory compounds such as volatile fatty acids (VFAs) [8,9]. As a result, the HS-AD process can be prone to frequent process inhibition, causing lower methane yields and restricting its widespread adoption. Efficient mass transfer within the high-solids substrate is a pivotal factor for facilitating microbial activity and ensuring a stable digestion process [10]. However, achieving effective mass transfer in HS-AD has proven to be a significant challenge, particularly when attempting to scale up the technology for industrial applications. Consequently, there is an urgent need to comprehensively understand and enhance the mass transfer processes within the HS-AD.

Fluid rheology plays a crucial role in dictating reactor mixing performance and mass transfer characteristics [11,12]. This, in turn, affects the selection of appropriate equipment for waste transportation and mixing within the reactor [13]. The rheological behavior of HS-AD markedly differs from that of LS-AD due to the high solid content, ultimately influencing mass transfer efficiency. Current studies suggest that the increase in TS content leads to a higher viscosity and consistency index [14]. The operating temperature of the digestion process also impacts the rheology of the digestate. Additionally, longer digestion times have been observed to decrease yield stress, which positively influences the pumping characteristics of the system. Given the heterogeneous polyphase nature of the HS-AD system, a comprehensive analysis of its rheological properties has become imperative to enhance the understanding of the fluid behavior and devise appropriate strategies for optimal mass transfer.

To address the limitations and challenges faced by HS-AD, process optimization and mass transfer enhancement have emerged as critical avenues of research, which can effectively mitigate the adverse effects of inhibitory compounds, enhance the rates of heat and mass transfer, and ultimately improve the biogas yield and substrate degradation efficiency [15,16]. One key aspect of process optimization involves addressing the limitations posed by the time required for the solubilization of the substrate particles in HS-AD. Pretreatments are commonly employed to facilitate the solubilization of the substrate particles. The mechanical and thermal pretreatments have shown promising results in enhancing the solubilization of the substrate [17]. In terms of operational considerations, effective mixing of the digester contents plays a crucial role in facilitating mass transfer [18], including the efficient utilization of digester volume, the prevention of stratification and formation of foam, scum, and crusts, the uniform distribution of pH and temperature, dispersion metabolic end products and any toxic materials present in the influent, the promotion of biogas release, and intimate contact between bacteria, bacterial enzymes, and their substrates [19]. Various methods, such as gas recirculation, liquid/slurry recirculation, and mechanical stirring, can be employed to maintain solids in suspension and promote a homogenous mixture [20]. Noteworthily, the development of membrane-based anaerobic systems has demonstrated potential in preserving biomass and enhancing the mass transfer between microorganisms and substrates [21].

Moreover, the addition of carbon-based materials has been explored, such as biochar, further improving mass transfer in HS-AD [22]. Biochar, given its various sources and low production cost, has emerged as a particularly promising material in this regard. The full potential of HS-AD technology for waste treatment can be released by enhanced mass transfer. However, the enhancement strategies of mass transfer in HS-AD have still not been systematically reviewed.

A comprehensive understanding of and mass transfer enhancement within the HS-AD system are necessary. Previous research has primarily centered on evaluating HS-AD, with a particular emphasis on process design and optimization, operational parameters, performance enhancement strategies, and mass transfer characteristics of specific processes in HS-AD. Nonetheless, a comprehensive evaluation of diverse approaches to boost productivity through increased mass transfer has yet to be conducted in HS-AD. The objective of this review is to bridge the existing knowledge gap by offering a comprehensive overview and examination of existing research on mass transfer phenomena, digester process design, and various enhancement methods (innovative pretreatment methods, effective mixing, and the addition of biochar), which are essential for unlocking the full potential of HS-AD as a sustainable waste management solution. This review can provide valuable insights for optimizing HS-AD and guide future research. Further research is required to systematically address and improve mass transfer in HS-AD, thus making it a more viable and efficient technology.

## 2. Overview of HS-AD and Process Design

### 2.1. Basics and Influencing Factors of HS-AD

HS-AD provides an efficient solution for the organic fraction of municipal solid wastes, encompassing food waste, agriculture waste, cow manure, sewage sludge, energy crops, municipal solid waste, garden waste, and industrial waste streams [23]. HS-AD is facilitated by distinct bacterial and archaeal communities under oxygen-depleted conditions, yielding nutrient-rich digestate along with around 60% methane (CH_4_) and around 40% carbon dioxide (CO_2_) as by-products [24]. The process of AD involves a series of interconnected biochemical and physicochemical reactions that occur both sequentially and simultaneously. These reactions can be classified into four stages: hydrolysis, acidogenesis, acetogenic, and methanogenesis [25]. Hydrolysis is the initial rate-limiting stage in AD. Hydrolysis bacteria break down complex organic compounds into simpler soluble substances, which are further metabolized during the fermentation step by microbes, leading to the production of VFAs. Acetic acid production, another critical step, is facilitated by genera such as *Syntrophomonas* and *Syntrophobacter*. Meanwhile, methanogens are divided into hydrogenotrophic and acetoclastic, which consume hydrogen and CO_2_ or utilize acetate as the primary substrate for methane production, respectively. Each phase is facilitated by specific microbial communities, and the interdependence and interconnectedness of the microbial communities between these stages is essential to maintain the balance of the microbial community and achieve optimal AD performance [26]. Disruptions or imbalances in the microbial community during these phases can result in the failure of AD systems [27].

Considering the influencing factors, such as organic loading rate (OLR), pH, the presence of inhibitory and harmful compounds, temperature, and solids retention time (SRT), is essential for maximizing HS-AD efficiency [28]. The OLR in HS-AD is approximately 10 VS/(m^3^·d), while the LS-AD exhibits an OLR of around 5–6 kgVS/(m^3^·d). Insufficient OLR leads to insufficient biogas production, and excessive OLR leads to organic overload and toxicity issues [29]. It is also essential to maintain the appropriate pH range (6.5–7.5) for the activity of acid-producing bacteria and methanogenic archaea [10]. Extreme pH values under 6.3 or over 7.8 could hinder the microbial species responsible for AD and negatively impact methane production [28]. The accumulation of VFAs during acidogenesis decreases the pH of AD systems and thus inhibits the methanogenic process of methanogens. Continuous monitoring and appropriate measures are necessary to manage toxic compounds such as ammonia, sulfides, heavy metals, and antibiotics that inhibit microbial activity and lead to process failure [4]. In addition, temperature is a crucial factor in AD, and it can be classified into three primary ranges: psychrophilic (10–30 °C), mesophilic (35–40 °C), and thermophilic (55–60 °C) [1]. The metabolic activity of microorganisms is temperature-dependent, with higher temperatures generally resulting in faster digestion. Additionally, SRT plays a significant role in preserving microbial balance and functional activity within the AD [30]. The longer SRT are often recommended to mitigate irregular organic loading and toxic compounds, but achieving optimal performance requires careful consideration of the interplay among SRT, organic loading, and specific system characteristics. Despite the challenges associated with maintaining the process stability of HS-AD, various strategies have been developed to address these difficulties. These strategies include substrate pretreatment, mixing optimization based on rheological properties, and the regulation of microbial populations involved in AD.

### 2.2. Classification and Commercial Systems of HS-AD

The design choice, implementation scale, and operation modes in digesters have a significant impact on the spatiotemporal hydrodynamics, biokinetics, as well as heat and mass transfer [1]. In the field of HS-AD, various reactor configurations have been developed to accommodate different operational requirements. These configurations include the flow orientation (horizontal and vertical), operating temperature (psychrophilic, mesophilic, and thermophilic), number of stages (single and multistage), and feeding mode (batch and continuous) [31]. The capacity of the HS-AD experienced a significant 50% growth between 2010 and 2015 in Europe, despite its utilization being limited to only treating 35% of the total waste processed by AD [32].

**Figure 1:**
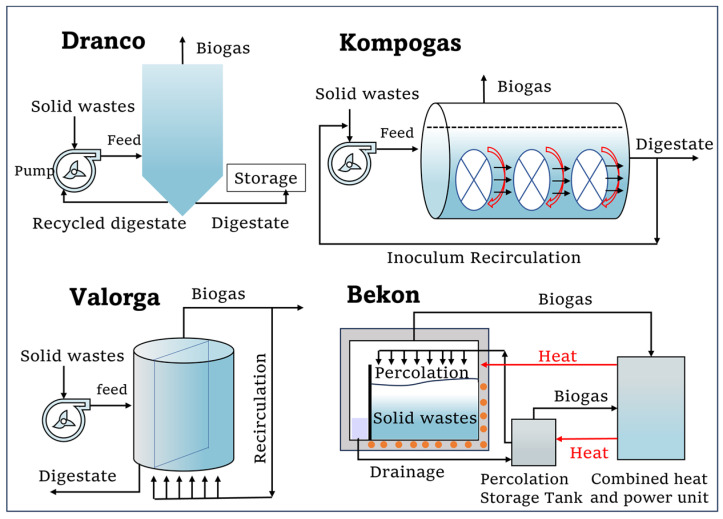
The classification of the HS-AD process technology.

Common commercial systems utilized in the HS-AD field include Dranco [33], VALORGA [34], KOMPOGAS [35], BEKON, Biocel, and BIOFerm (Figure 1) [30]. The raw materials contain TS in the range of 30–40%. Around 60% of the overall capacity for municipal waste treatment in Europe is attributed to HS-AD systems [36]. The operational strategies adopted by each of these systems in designing digesters are evaluated next to overcome the major challenges (slow mass transfer) in HS-AD. The Dranco, VALORGA, and KOMPOGAS systems are all typical continuous and single-stage HS-AD systems, which can be operated continuously for solid contents of between 20 and 60% [7,37]. It is worth noting that some of these dry systems lack internal mixing, and therefore, substrate and digestate are mixed before feeding the digester [35]. The Dranco system (thermophilic or mesophilic) has no mixing apart from that occurring due to the downward plug flow of the feeding waste. A Dranco digester in Brecht, Belgium, treated biomass feedstock with TS = 35% (15% food waste, 75% garden waste, and 10% paper) for HRT = 14 days to produce 468 m^3^/t of biogas. The VALORGA system (thermophilic or mesophilic, vertical) uses pressurized biogas for mixing, but the process of pressurizing biogas necessitates a significant amount of energy, and there is a notable likelihood for organic materials to obstruct the nozzles [24]. The KOMPOGAS system (thermophilic, horizontal), which originated in Switzerland in the 1980s, can complete digestion in approximately 14–20 days by utilizing a horizontal push flow and internal stirring paddle for mixing. The operation of this digester falls within a range of 23% to 28% total solids, which resembles the methodology employed in the Dranco process [24]. Compared to the continuous HS-AD systems above, the batch HS-AD systems were developed relatively later. Batch HS-AD systems reduce system complexity and machinery maintenance requirements [38]. The BEKON system in Germany, Biocel system in Netherlands, and BIOFerm system (single-stage, mesophilic) were shown to be able to treat wastes with a feed TS of 25% to 35% at HRT = 28 days, but the process stability (incomplete mixing and accumulation of VFAs) of the batch-mode digesters remained [39]. More in-depth information can be found in previous studies [1,30,40].

As well as the above standalone digesters, common multistage HS-AD has been leveraged to enhance the overall process efficiency. The initial phase involves the processes of hydrolysis and acidogenesis, whereas the subsequent stage is accountable for acetogenesis and methanogenesis. A novel biogas production method called Biopercolat was devised, employing a two-step anaerobic digestion process, including a hydrolysis chamber with axial mixing and a methanogenic chamber [41]. The Biotechnische Abfallverwertung (BTA) system utilizes a hydrocyclone for solid or liquid separation after which the solid fraction is mixed with pretreated leachate and then pumped into a hydrolysis tank [42]. The dual-stage digester configuration facilitates a faster attainment of stability compared to single-stage reactors [43]. Nonetheless, there are certain drawbacks associated with utilizing a two-stage system, including the accumulation of hydrogen that can impede the growth of acid-forming bacteria, reduced stability of biomass, technical complexity, and storage limitations, as well as increased operational expenses [44].

The adoption of HS-AD technologies incurs capital and operational costs, which vary depending on the technology chosen. For example, companies implementing Dranco technology in Saffenburg (Austria) and Brecht (Belgium) with large capacities require significant investments in digester capacity, maintenance, and heat energy to maintain thermophilic temperatures [30]. BEKON technology, with its advantages of not requiring pumps and agitators, and no need for pretreatment of bulk waste, proves to be more cost-effective in terms of machinery and operational costs, but it has limited digestion efficiency and a large reactor volume [38]. Detailed economic analyses are necessary to evaluate the cost-effectiveness of adopting these technologies, and cost estimations can be obtained from simulation models. Furthermore, technological advancements to increase the methane content in biogas may potentially reduce the overall cost of the HS-AD process, enhancing its economic viability.

Several case studies in the systematic review provided practical data on anaerobic digestion processes. For instance, Fernández et al. [45] observed that a TS content at 20% in the reactor yielded significantly better methane production compared to a TS content at 30%. Rodriguez et al. [46] identified the optimum solids retention time of 20 days for dry mesophilic anaerobic digestion of OFMSW. The data of HS-AD technologies offer promising solutions for efficiently treating high solid wastes. The prevalence of single-stage and continuous digesters is higher; however, the successful implementation of HS-AD processes in industrial settings relies on factors such as substrate properties, site choice, and size, as well as efficient automation for controlling process parameters.

## 3. Rheological Characteristics and Limitation of Mass Transfer of HS-AD

**Figure 2:**
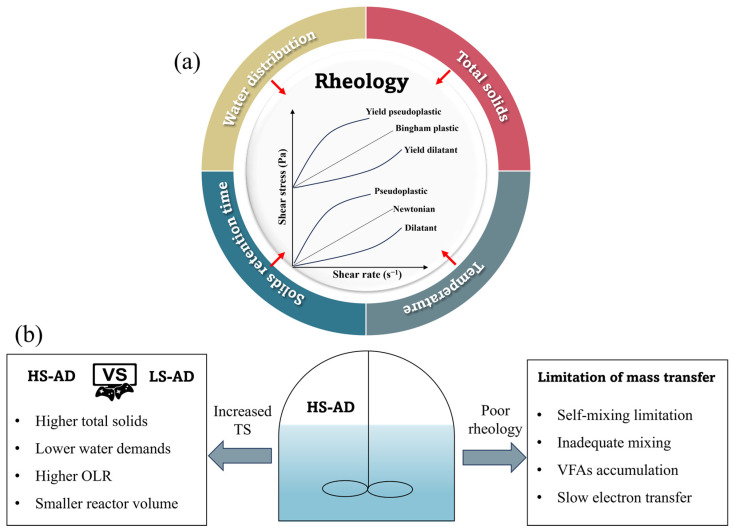
Rheological characteristics and their influencing factors of digestate (**a**) and limitation of mass transfer in HS-AD (**b**).

Rheological properties (e.g., yield stress and viscosity) are of fundamental importance to HS-AD multimedia systems because they have a strong influence on the overall hydrodynamics of mixing, mass transfer efficiency, and the dynamics requirements of anaerobic digesters. Based on the complex rheological properties, HS-AD suffers from a series of severe limitations (Figure 2), which hinder its widespread application. Identifying the system rheological properties and mass transfer limitations is pivotal for optimizing methane production.

### 3.1. Rheological Characteristics of Digestate

According to the characteristics of shear stress change at different shear rates, fluids with yield stress can be classified as yield-pseudoplastic, Bingham plastic, and yield-dilatant fluids, while fluids without yield stress are pseudoplastic, Newtonian, and dilatant fluids [47]. Yield-pseudoplastic and pseudoplastic fluids have an upward convex curve, yield-dilatant and dilatant fluids have a downward concave curve, and Bingham plastic and Newtonian fluids have a linear relationship. The non-Newtonian fluid nature of HS-AD slurry means it has shear-thinning, thixotropic, and viscoelastic properties. The rheology of non-Newtonian fluids cannot be described by a single value of its viscosity (defined as the ratio between shear stress τ and shear rate γ). With the increased TS, the viscosity of high-solids digestate decreases with increasing shear rate [6,47,48]. The rheological model to characterize the rheological behaviors of different types of digestate was employed in previous studies [10]. The most basic and common rheological models include the power law [49], Bingham [12], and Herschel–Bulkley [50,51]. The Herschel–Bulkley model was often employed to provide valuable insights into the flow characteristics of digestate [12]. During high shear conditions, the applied force induces changes in the sludge structure over time, and the thixotropic behavior becomes apparent if the applied force is kept within the deformation limit of the digestate flocs, resulting in the formation of a viscosity hysteresis loop upon force removal. Storage modulus (G′) and loss modulus (G″) of the digestate characterize its elastic properties and viscous properties, respectively. The presence of a higher TS content correlated with an enhanced resistance to deformation, as evidenced by elevated values of G′ and G″ [48]. A comprehensive understanding of the rheological properties involved is crucial for optimizing the performance and design of HS-AD systems. However, the validity of standard rheological measurements for plane-to-plane, cone-to-plane, or cylindrical rotational rheometers is compromised by the fact that the heterogeneity of high-solids digestate leads to slippage during the measurement process [52]. Therefore, methods and tools for characterizing digests still need to be further explored.

Several studies on the rheological behavior of HS-AD focused on TS content [53], temperature [54], moisture distribution [48], and SRT [6], especially for anaerobically digested sludge (Figure 2a). Higher TS levels lead to an increase in flow resistance and thixotropic kinetic coefficient. Exceeding a critical threshold of 7% TS can lead to a significant increase in infinite viscosity and yield stress, making digestate transport difficult [6,55]. Additionally, the relationship between shear stress and TS concentration can be represented by either exponential or power functions [5]. However, the exponential model tends to overestimate shear stress at high TS concentrations, while the power function underestimates it. Moreover, temperature conditions and moisture distribution affect the flowability of digested sludge. The digestate viscosity at mesophilic temperatures is relatively lower compared to that at thermophilic temperatures [56]. A higher temperature offers better flowability due to a higher proportion of free and interstitial water, while surface water is more prevalent in mesophilic systems. The higher surface water may lead to poor rheological properties [48]. Moreover, the rheological properties of digested sludge are significantly affected by SRT, and the SRT in HS-AD is inversely associated with the shear stress, viscosity, and yield stress [6]. Among the various factors affecting rheological properties, the presence of solids in the fluid is thought to be a key factor, and more significant than temperature [24]. Despite poor kinetic properties leading to slow mass transfer efficiency, it is difficult to quantitatively describe various factors that influence rheological properties and mass transfer.

### 3.2. Limitation of Mass Transfer

Unlike traditional LS-AD, the low water content of HS-AD hinders the efficient conversion of organic substrates into valuable biogas and impacts the transport of crucial substances, such as substrates and gases, and the distribution of nutrients (Figure 2b) [10]. Due to the high viscosity and rheology of solid substrate, conventional mixers fail to adequately ensure uniformity and may result in insufficient interaction between microorganisms and substrates. When the TS content exceeds 15% and agitation is inadequate, substrates may require reactions and diffusion to become accessible for further degradation by the inoculum. Insufficient mixing may impede reaction kinetics, causing mass transfer limitations that create areas where degradation occurs less efficiently, ultimately hindering microbial metabolism.

**Substrate and gas diffusion (self-mixing) limitation.** The diffusion of the substrate into the microorganisms may be limited, especially in HS-AD characterized by high solids content or dense microbial populations [22]. The formation of dense microflora restricts the diffusion of substrates and hinders the penetration of nutrients and gases into the core of these aggregates. Similarly, the diffusion of gases, such as CO_2_ and hydrogen, in HS-AD could be restricted, leading to gas accumulation in specific areas and hampering their utilization by microorganisms [1].

**Inadequate mixing.** Inadequate mixing can create localized areas of the over-concentration or under-concentration of substrates and microorganisms, leading to uneven reaction rates and potential process imbalances [57]. Poor rheological characteristics could potentially have a negative impact on the mixing effectiveness and energy consumption of the HS-AD procedure [58].

**VFAs accumulation.** The composition and diversity of microbial communities involved in HS-AD can also influence mass transfer limitations. One of the significant challenges in HS-AD lies in the accumulation and degradation of VFAs [2]. VFAs’ degradation is thermodynamically unfavorable under the standard state [59]. Only continuous consumption of hydrogen by methanogens is necessary to ensure the normal operation of the reaction. When VFAs’ concentrations exceed a certain threshold, they become toxic to methanogens (in particular, acetoclastic methanogens were more susceptible to this toxicity than hydrogenotrophic methanogens), leading to further process imbalances and reduced biogas production [60].

**Slow electron transfer between microorganisms.** Moreover, certain microorganisms involved in syntrophic interactions rely on efficient interspecies electron transfer (IET) mechanisms to exchange electrons and carry out metabolic processes cooperatively. IET encompasses interspecies hydrogen transfer (IHT) and interspecies formic acid transfer (IFT) [22]. In anaerobic environments, the transfer of H_2_ and HCOOH typically occurs through diffusion. However, this mode of transfer is characterized by sluggishness and vulnerability to electron loss due to compound leakage during mass transport [61]. Most hydrogenotrophic methanogens also utilize formic acid and hydrogen as electron donors. If the IET pathway is hindered, the accumulation of VFAs could occur to further impact microbial metabolism and biogas production.

## 4. Strategies for Enhancing Mass Transfer in HS-AD

**Figure 3:**
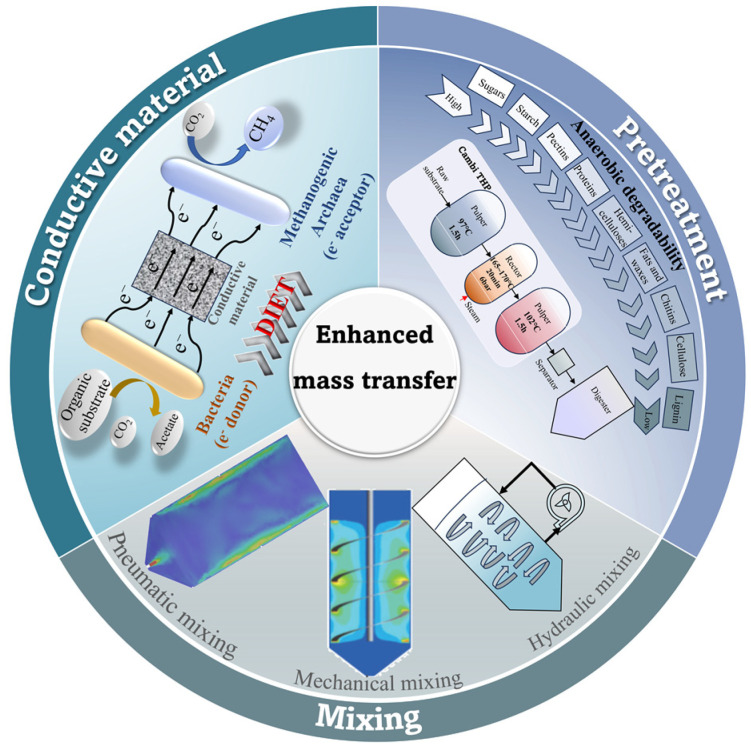
Strategies for enhancing mass transfer in HS-AD, including substrate pretreatment, mixing, and conductive material addition.

Two major challenges in the mass transfer enhancement of HS-AD are inefficient substrate hydrolysis and limited microbial accessibility to the substrate. To overcome mass transfer limitations, various effective strategies could be employed, involving the improvement in substrate diffusion characteristics and mixing optimization, and the maintenance of efficient electron transfer in microbial community balance (Figure 3 and Table 1). The full potential of the HS-AD process can be unleashed to maximize biogas production from OFMSW.

### 4.1. Substrate Pretreatment

The limited biodegradability and slow hydrolysis rate of substrates (e.g., cellulose, lignin, and fats) have been found to be significant challenges during the processing of organic wastes. To address these challenges, substrate pretreatment has emerged as an effective strategy to improve substrate biodegradability and the hydrolysis rate of HS-AD [2]. After undergoing pretreatment, the solubilization of substrates exhibits a logarithmic increase, while its elastic modulus in the linear viscoelastic range undergoes a logarithmic decrease. Substrate pretreatment improves the rheological properties (a decrease in viscosity) and weakens the mass transfer resistance. These pretreatment technologies include mechanical pretreatments, thermal pretreatment, chemical pretreatment, biological pretreatment, and combined pretreatment [77]. Biological pretreatment requires a long time for processing. The significant barriers preventing the widespread implementation of chemical pretreatment include substantial capital expenses, extensive energy consumption, the need for specific chemicals, and complex operational requirements (such as maintenance and odor control). The application of both mechanical and thermal pretreatments has gained significant traction in industrial settings. Mechanical and thermal pretreatments offer distinct advantages in enhancing mass transfer and optimizing the digestion performance of various organic wastes. Among the widely reported pretreatment methods tested at lab scale, only a few mechanical, thermal, and thermochemical methods were successfully applied at full scale [78]. Therefore, this section focuses on summarizing the status of mechanical, thermal, and biological pretreatment (Table 1).

**Mechanical pretreatment.** Mechanical pretreatment, including mechanical shredding, sonication, liquid shear, high-pressure homogenization, and others [10], can reduce the particle size of substrates; various aspects crucial for the physical properties of substrate and microbial conversion are affected, such as microbial growth and specific surface area, as well as heat and mass transfer [1]. The microbial transformation initiates from the exterior and penetrates the innermost region of the surface. Particle downsizing has been observed to improve process stability in HS-AD by increasing mass transfer and metabolite distribution, thus facilitating better access of biomass and metabolites to microbes [79]. Mechanical processes such as stirred ball mills (SBM) and high-pressure homogenizers (HPH) have been used to effectively rupture the cell walls of the feedstock within minutes [80]. Compared to the untreated substrates, mechanical pretreatments lead to a significant enhancement of 20–40% in methane production with the particles up to ≤30 mm in particle size [77]. The previous research revealed that reducing the particle size from 12.7 mm to 1.0 mm resulted in a significant enhancement of methane production by up to 26.4% during dry anaerobic digestion of stored corn stover [66]. The rate of hydrolysis and digestion performance in AD are significantly influenced by particle size and composition [81], and relevant substrates may exhibit varying optimal particle sizes. For example, the reduction in particle size has been demonstrated to significantly enhance biogas production for substrates characterized by high fiber content and low degradability [82]. While fine milling improves substrate accessibility and enhances the conversion rate [83], the risk of acidification is also increased due to the rapid production and accumulation of VFAs. Therefore, enhancing the performance of HS-AD by utilizing smaller waste biomass particles may not always be necessary. A comprehensive understanding of the particle distribution curve and digestion performance for diverse substrates, such as cattle manure, roadside grass, and corn stover, can provide valuable insights into determining distinct optimal particle sizes in HS-AD [84]. However, comprehensive information on the effect of particle size on biogas and methane production is lacking. In addition, the utilization of a higher energy input and increased TS concentration can enhance the efficiency of substrate disintegration. To achieve a maximum sludge disintegration degree, energy consumptions of 8450, 5351, and 3252 kJ/kg TS were required for sludges with TS concentrations of 10, 15, and 25 g/L, respectively [85]. The HPH pretreatment resulted in a lower energy consumption compared to the energy produced, resulting in a positive net energy balance. At pressures of 10 MPa, the HPH pretreated sludge exhibited positive energy values of 790 kJ/kg TS, respectively, compared to the control group (290 kJ/kg TS) [86].

**Thermal pretreatment.** Thermal pretreatment involves exposing substrates to elevated temperatures to promote chemical reactions and break down complex biopolymers and organic matter into simpler, more bioavailable compounds. This transformation results in a reduced particle size and increased surface area, making the substrates more accessible to microbial activity during the subsequent HS-AD. Thermal pretreatments are typically categorized into low-temperature pretreatment (60–100 °C) and high-temperature pretreatment (100–180 °C) based on the temperature range [87]. However, the practical implementation of increased biogas production is hindered due to the higher energy consumption required for the high-temperature thermal treatment. The energy balance of thermal pretreatment depends on factors such as the pretreatment temperature, TS concentration, and the efficiency of heat recovery and utilization. An 80 °C pretreatment was energetically feasible compared to 100 and 200 °C [87]. The net electricity balance under the 80 °C pretreatment (1390 kJ/kgTS) increased by about 30% more than the control group (1070 kJ/kgTS) [88]. The biodegradability of substrates is enhanced through thermal pretreatment via two primary mechanisms: the production of biodegradable organic substances through thermal hydrolysis, and the liberation of intracellular organic substances following the disruption of cellular walls and the membranes’ exposure to high temperatures [89]. Additionally, thermal hydrolysis leads to the decomposition of triglyceride-rich substrates, generating a series of VFAs, such as acetic, propionic, butyric, and valeric acids [90]. The application of a thermal pretreatment at a temperature of 70 °C for a duration of 3 days was implemented to improve the dry AD process of swine manure in a previous study [91]. Remarkably, the introduction of this pretreatment resulted in an astounding 390% increase in methane production (416 mL CH_4_/g VS) compared to the untreated feedstock. Thermal pretreatment has been extensively investigated for waste-activated sludge, but other applications with various substrates in HS-AD, such as manure and lignocellulosic, need to be studied further in the future.

**Biological pretreatment.** Biological pretreatment involves the utilization of fungi, bacteria, enzymes, and microbial consortia to enhance methane production and cellulose recovery while minimizing reliance on harsh chemicals and extreme temperatures. Fungi have demonstrated their proficiency in generating enzymes that facilitate the biological pretreatment process [92]. The optimal temperature range for these fungi is 28 to 37 °C over a duration of 12 days to 8 weeks. To ensure effective fungal pretreatment, it is imperative to autoclave the feedstock prior to inoculation and provide an aerated environment. Furthermore, enzymatic pretreatment, a key facet of biological pretreatment, harnesses the potential of enzymes such as laccase, pectinase, cellulase, hemicellulase, and β-glucosidase to enhance methane production [93]. Notably, this enzymatic approach thrives in both aerobic and anaerobic conditions, effectively boosting hydrolysis during AD. The enzymatic transformation is particularly observed in studies conducted under mesophilic thermal conditions, maintaining temperatures at 37 °C for durations spanning 4 to 24 h. The microbial consortium pretreatment emerges as a dynamic avenue, involving the orchestration of diverse microbial agents such as cellulytic bacteria, yeast, and specific fungal species. These consortia operate optimally within temperatures ranging from 20 to 55 °C for durations of 12 h to 20 days. The utilization of domesticated paddy soil microbes was employed for the pretreatment of rice straw and pig manure co-substrates, enhancing the hydrolytic acidification process of the co-substrates with a total solid content of 20%, resulting in a significant reduction in methane production time by 43.4% [94]. Biological pretreatment strategies offer advantages in terms of cost-effectiveness and reduced reliance on extreme conditions [95]. However, their careful assessment is necessary due to challenges such as longer treatment times, larger space requirements, and the susceptibility of complex compounds to the process.

Substrate pretreatment holds immense promise for overcoming the challenges faced in HS-AD processes. As for OFMSW, mechanical pretreatment methods are fully utilized. The Thermal Hydrolysis Process (THP) has been fully applied in only a few examples of sludge treatment, such as the Cambi, Porteous, and Zimpro processes [96]. In addition, a coupling combination of chemical and mechanical pretreatment improves the overall process efficiency by reducing cost and energy consumption [77]. However, the challenges of these technologies involve developing novel pretreatment methods and optimizing the combinations between different pretreatments in the future.

### 4.2. Mixing Optimization

Achieving the homogeneity of substrates, microorganisms, and gases throughout HS-AD presents a significant challenge due to the high viscosity and rheology of the solid wastes, coupled with the absence of specific mixers [5,97]. Moreover, poor mixing can hinder heat and mass transfer, leading to further poor diffusion of intermediate metabolites and exacerbating process difficulties [30,55]. Effective mass transfer relies on proper mixing, which enables close contact between substrates and microorganisms, ensuring efficient heat transfer and the release of gaseous products [57]. Mixing strategies and designs to improve mass transfer efficiency is critical to the stable and efficient operation of the HS-AD system. However, achieving effective mixing in HS-AD systems with 20% TS or more can indeed pose a challenge. The presence of a high TS content leads to the formation of a thicker and more viscous substrate, thereby making mechanical mixing more arduous and energy-intensive. Inefficient mixing can result in uneven decomposition and localized accumulation of volatile fatty acids, which may have detrimental effects on biogas production. Consequently, some operators choose less aggressive mixing strategies such as intermittent mixing or employ passive mixing systems such as inclined or vertical digesters in AD systems characterized by a high TS content. These approaches not only reduce energy consumption but also provide adequate mixing support for the digestion process.

**Inoculum addition.** Inoculum addition focuses on achieving good homogenization of fresh inoculum with substrate within the reactor, enhancing adequate inoculation, and preventing overloading and particle sedimentation [98,99]. By introducing active microbial consortia into the reactor, inoculum addition plays a critical role in kickstarting the HS-AD.

**Mixing methods.** Mixing methods can be classified as mechanical, pneumatic, or hydraulic (Table 1). Mechanical mixing is driven by stirring blades, and its mixing efficiency depends on factors such as the stirring paddle design, bottom clearance, paddle clearance, and substrate rheology. Pneumatic mixing involves the injection of air or biogas into the digester to induce turbulence and promote material convection and circulation [18,100]. Hydraulic mixing is accomplished by recirculating the HS-AD slurry through an external pump to mix the material [30,101].

Mechanical mixing is widely used in digestion reactors [20]. The specific paddle designs could significantly improve biogas production [102,103]. Compared to a single turbine propeller, screw propeller mixing results in a superior shear force and viscosity distribution, leading to over a 50% increase in biogas production of HS-AD [104]. The use of a single blade propeller in AD is not recommended due to the uneven distribution of shear force resulting in high stress near the blade and dead zones near the wall. A comparative assessment of the mixing effects of turbine agitators with four, six, and marine propeller blades during the acidification and fermentation of lignocellulosic wastes showed that the clearance between the impeller and the bottom has a significant effect on the slurry flow pattern [105].

Compared to mechanical mixing, pneumatic mixing requires fewer moving parts and complex components [106]. This method has been commercialized in gas-mixed digesters, such as VALORGA. In addition, the nanobubble technology has been demonstrated to increase the mobility of water in the surrounding aquatic environment, facilitating a higher transfer of nutrients to microbial cells, and thereby increasing biological activity and enzyme activity [107].

In the realm of HS-AD applications, two distinct approaches prevail: continuous and batch systems. In continuous setups, a constant stream of organic waste is introduced into the digester, ensuring an ongoing biogas harvest. These systems necessitate efficient mixing for stability and sustained biogas production, making them ideal for large-scale industrial contexts. The key determinant of biogas and methane production in continuous systems is the OLR, which can be adjusted while preserving process stability. Mixing mechanisms play a crucial role in these systems. VALORGAS utilizes biogas recirculation at the bottom of the AD for efficient mixing. KOMPOGAS employs a slow internal axial rotation for mixing, while Dranco AD relies on external mixing, with internal mixing being more energy-intensive. On the other hand, batch AD systems involve loading a finite amount of organic material into the digester, with digestion occurring in distinct cycles. Mixing is less critical in batch systems since the substrate is well-mixed during loading. After each batch, the digester is emptied and a new one is loaded. Batch systems are commonly found in small-scale or decentralized AD applications where mixing demands are lower. For instance, BEKON in Harsewinkel, Germany, employs a garage-type reactor, using 50% of the digestate as inoculum to initiate new batch cycles [108]. In general, the selection between continuous and batch AD systems is contingent upon specific operational requirements, scale, and available resources. Continuous systems are particularly suitable for industrial settings with high biogas demand, whereas batch systems are more appropriate for smaller-scale decentralized applications with lower mixing prerequisites. Additionally, the choice of mixing mechanism significantly influences system efficiency and operational costs.

During the recirculation process, soluble organic compounds undergo partial transfer from the solid phase to the liquid phase, thereby mitigating the substantial resistance encountered by mass transfer and gas diffusion within the solid phase [109,110]. Liquid digestate recirculation is also used to dilute the inhibitory effect of accumulated ammonia, VFAs, and other metabolites in liquid digestate, further enhancing the mass transfer in HS-AD between the inoculum and substrate [101]. However, the efficiency of hydraulic mixing is limited compared to mechanical and pneumatic mixing.

**Mixing strength and mixing time.** The effect of mixing strength and mixing time significantly impacts the overall biogas productivity [57,111]. Minimizing energy consumption while achieving optimal mixing is a major challenge. The typical mixing power input of 5–8 W·m^−3^ for AD was recommended by the U.S. Environmental Protection Agency; however, for HS-AD, determining the optimal mixing power input remains a topic of debate. Mixing energy consumption increases with rising TS content. It is important to note that simply supplying more energy to the mixing system does not necessarily lead to improved mixing performance and may even negate gains in the energy production process. Balancing mixing intensity and time is crucial to maximize biogas productivity while minimizing energy consumption.

Indeed, vigorous mixing may easily lead to the destruction of microbial floc and the accumulation of VFAs, negatively affecting biogas production rates [5,112]. With the increase in mixing energy input, the colloidal force and network strength of digestate flocs were weakened. In terms of mixing time, intermittent mixing was found to increase methane yield compared to continuous mixing in laboratory-scale and pilot-scale digestion reactors [5,69]. Intermittent mixing promoted the conversion of complex substrates to soluble organic matter in HS-AD [113], and had the maximum acidification, acetylation, and methanogenesis efficiencies [114]. However, the direct relationship between specific electricity consumption and active digester volume and dry matter remains unclear due to variations in impeller geometry and substrate properties. The optimization of mixing energy demand can be achieved through adjustments in mixing time and design. For example, by strategically adjusting the positioning of agitators within the digester, it is possible to achieve a 50% reduction in energy consumption for mixing without compromising operational efficiency [115]. Similarly, through the optimization of mixing time, an impressive 85% decrease in electric power consumption was achieved by the pilot-scale digester [116].

Various mixing methods and strategies have been employed to enhance mixing efficiency in both conventional and HS-AD processes. Although mechanical mixing has been most widely adopted in various digesters, the selection of the mixing mode should be based on substrate properties, slurry rheology, reactor and impeller geometry, and feed rate. Generally, intermittent mixing is recommended [5,63]. Achieving the optimal mixing strategy remains a challenging task. Computational fluid dynamics (CFD) is a highly valuable tool for predicting fluid flow and has the potential to determine effective mixing strategies for digesters of varying scales [97]. CFD offers an efficient and robust solution at a significantly lower cost compared to experimental studies. The current range of modeling tools and computer capabilities can be effectively utilized for simulating and optimizing mechanical, pneumatic, and hydraulic mixing optimization. Successful applications of CFD include designing digester configurations, selecting optimal mixing conditions, and in particular, simulating mixing time based on substrate characteristics [117]. However, the application of CFD in HS-AD is limited to fluid dynamics and does not fully incorporate biokinetic models (e.g., Anaerobic Digestion Model 1, ADM1), which may constrain its ability to accurately predict actual reactions. Therefore, future research should strive to establish a correlation between predicted parameters obtained from CFD simulations and biological impacts with the help of similar tools such as artificial neural networks.

### 4.3. Biochar Addition

Biochar is a carbon-rich material produced from the pyrolysis of organic waste. Its unique porous structure and high specific surface area provide an ideal environment for microbial colonization and activity, demonstrating significant potential in promoting the performance of anaerobic microbes and enhancing overall AD efficiency (Table 1). The addition of biochar resulted in methane contents ranging from 88.5% to 96.7%, whereas the control reactor had a methane content of only 67.9%. Optimal performance was achieved with a 15 g/L biochar dosage and a 3 g substrate/g inoculum organic load rate [118]. Mass transfer enhancement by biochar in HS-AD is a topic of increasing interest and importance in the field of environmental biotechnology.

The surface properties and porous structure of biochar exhibit a strong adsorption capacity. Biochar possesses abundant functional groups, such as hydroxyl, carboxyl, and phenolic groups, which facilitate strong interactions with the organic compounds and nutrients present in digestate [59]. The well-developed pore network of biochar provides a physical pathway for the diffusion of gases and soluble compounds, facilitating their transport to and from microbial cells [119,120]. The adsorption capacity of biochar facilitates the removal of metabolic by-products and reduces the negative impact of these inhibitory substances on anaerobic microbial communities [121]. Notably, biochar can serve as a buffer against sudden changes in pH, temperature, and other physicochemical parameters, providing a more stable and favorable environment for microbial communities [122]. The buffering capacity of biochar is primarily influenced by the pyrolysis temperature, with a decrease in acidic functional groups (from 4.17 to 0.22 mmol/g) and an increase in alkaline functional groups (from 0.15 to 3.55 mmol/g) when the pyrolysis temperature increased from 200 to 800 °C [122]. By minimizing the impact and buildup of inhibitory substances, biochar ensures consistent microbial activity and methane production, contributing to the sustained and efficient operation of AD processes.

The abundant mesoporous structure of biochar could promote the aggregation and retention of a wide range of microorganisms, including syntrophic bacteria, fermenters, and methanogens, to foster the development of a diverse and robust microbial community [123,124,125]. Microbial cells tend to adhere to biochar surfaces due to electrostatic interactions and the availability of favorable microhabitats. The aggregation and spatial organization of microorganisms creates a locally concentrated ecological environment on the biochar surface, which favors interspecies interactions and promotes the formation of microbial consortia with syntrophic metabolic capabilities, thus facilitating the establishment of a symbiotic relationship between these bacteria and methanogens [120]. Under varying operating conditions and substrate compositions, the ability of biochar to support a diverse microbial consortium enhances the system’s adaptability and efficiency, making it less susceptible to disturbances and ensuring sustained methane production [23,122].

Furthermore, the utilization of biochar can serve as a facilitator for electron transfer in HS-AD, thereby establishing an optimal setting for the effective exchange and transmission of electrons, further contributing to elevated mass transfer [126]. The conductive properties of biochar enable it to promote electron shuttling between different microbial species via a direct interspecies electron transfer (DIET) pathway, and to enhance syntrophic interactions among microorganisms [10,127]. In previous studies, the use of Fe_3_O_4_-modified water hyacinth biochar (Fe_3_O_4_/WHB) resulted in a remarkable 60% increase in methane production through an enhanced DIET [128]. Novel carbon-based nanoparticles were found to improve the efficiency of mass transfer and reduce the presence of inhibitors in a previous study [129,130]. In addition, the overall expense associated with biochar, depending on the type of raw material, pyrolysis technique, and activating agent, may vary from 0.2 to 0.5 USD/kg. The biochar is more cost-effective compared to granular activated carbon, with prices ranging from 0.6 to 20 USD/kg [131]. The addition of biochar could potentially be economically viable for enhancing the thermophilic anaerobic digestion of food waste.

Mass transfer enhancement by biochar in AD is a multifaceted phenomenon that arises from its adsorption capacity, porous structure, ability to support microbial aggregation, and role in mediating electron transfer. Moreover, the stabilizing effect of biochar on AD processes ensures consistent and efficient methane production, making it a promising and sustainable strategy for improving digester performance. The characteristics of biochar directly affect its effectiveness in a complex HS-AD system. Future research in this area should focus on optimizing biochar properties, selecting the appropriate biochar variant, and incorporating it into various AD systems to further explore its potential for enhancing mass transfer and enhancing overall process efficiency. The utilization of diverse biomass sources and the adjustment of pyrolysis conditions enable the production of various biochar types.

## 5. Summary and Future Trends

HS-AD of OFMSW is a promising and efficient bioprocess for sustainable waste management and renewable energy generation. The design and selection of appropriate digesters play a vital role in maximizing process efficiency and methane production in HS-AD. However, HS-AD faces challenges related to mass transfer limitations that hinder the efficient conversion of organic substrates into valuable biogas. To address these challenges, substrate pretreatment has emerged as an effective strategy to improve biodegradability and hydrolysis via HS-AD. Efficient mixing techniques and equipment are vital to ensure the uniform distribution of substrates and microorganisms within the digester, thus enhancing mass transfer efficiency. Additionally, biochar enhances mass transfer and promotes the growth of diverse and resilient microbial communities in AD through its adsorption capacity, porous structure, ability to facilitate microbial aggregation, and role in mediating electron transfer.

The achievement of efficient mass transfer and homogenization within HS-AD poses a significant technical challenge. As the content of TS increases, the yield stress of anaerobically digested solid waste exhibits an exponential rise, thereby impeding mass transfer. This heightened yield stress not only restricts nutrient availability to microbes but also escalates energy consumption during substrate homogenization. Addressing this challenge requires extensive research into the rheological behavior within HS-AD digesters and careful optimization of TS conditions. From an economic perspective, methane production significantly impacts cost-effectiveness in HS-AD. Unfortunately, technical difficulties often result in reduced methane production, which jeopardizes economic viability. Therefore, strategies must be developed to enhance HS-AD processes, with a particular focus on lignocellulosic materials that hold immense potential for methane production. Overcoming their recalcitrance through appropriate pretreatment methods is crucial. Additionally, it is imperative to acknowledge that the HS-AD process operates at a comparatively slower pace in comparison to thermochemical methods such as pyrolysis and incineration, primarily due to the sluggish metabolic rate of anaerobic microbes. Consequently, meticulous consideration of the operational parameters and careful selection of the inoculum, particularly for lignocellulosic materials, are essential. Moreover, suitable pretreatment methods are required to effectively break down the intricate lignocellulosic structure.

Future trends in HS-AD involve the incorporation of innovative reactor designs, substrate pretreatment, the use of advanced modeling and simulation techniques, and the novel conductive materials to ensure the sustainability and success of HS-AD for OFMSW. The development of membrane-based anaerobic digestion systems has shown promise in selectively retaining biomass and enhancing the contact between microorganisms and substrates [132]. Each substrate pretreatment technique presents its own set of advantages and limitations. It is beneficial to enhance the synergy between different pretreatment methods for further optimization of methane production. In-depth modeling and simulation (such as CFD, ADM1, and artificial neural networks) of mass transfer phenomena have the potential to provide valuable insights into the fluid and biological dynamics of mass transfer in complex HS-AD systems. These models can aid in optimizing digester configurations, identifying potential bottlenecks, and predicting the performance under varying operating conditions. CFD-predicted parameters should be linked to biological impacts in future research. The utilization of novel and stable carbon-based nanoparticle materials with a substantial surface area has the potential to enhance biochar adsorption and accelerate microbial community bonding, thereby increasing digestion efficiency.

## Figures and Tables

**Table 1:** Performance of various strategies for enhancing mass transfer used in recent studies.

Strategies for Enhancing Mass Transfer		Operating Conditions		Substrates	AD Efficiency	Ref.
Substrate pretreatment	High-thermal pretreatment	140 °C for 3 h		Dewatered sludge	Increase in CH_4_ yield at 81%	[62]
	High-thermal pretreatment	121 °C at 103.4 kPa for 30 min		Food waste and cattle manure	Increase in CH_4_ yield at 139%	[63]
	Low-thermal pretreatment	70 °C for 3 days		Swine manure	Increase in CH_4_ yield at 39.5%	[64]
	Low-thermal pretreatment	60 °C for 3 h		Dewatered sludge	Increase in CH_4_ yield at 547%	[65]
	Mechanical pretreatment with grinding/chopping	Using a Wiley Mill, 1, 12.7 mm particle size		Corn stover	Increase in CH_4_ yield at 26.8	[66]
	Mechanical pretreatment with grinding/chopping	Using a cutting mill, 2.0, 1.0, 0.5, 0.25, 0.12 mm particle size		Wheat straw	Increase in CH_4_ yield at 37.8	[67]
	Enzyme	peroxidase; 6 h–24 h; 30 °C		Corn stover	Improved lignin degradation at 15%	[68]
Mixing optimization	Mechanical mixing	Mixer type: stirrer	Mixing interval: continuous	Cattle manure	Biogas production rate at 0.67 L/L	[69]
			Mixing interval: minimal		Biogas production rate at 0.75 L/L	
			Mixing interval: intermittent		Biogas production rate at 0.68 L/L	
		Mixer type: impeller	Mixing interval: continuous		Biogas production rate at 1.20 L/L	
			Mixing interval: minimal		Biogas production rate at 1.21 L/L	
	Pneumatic mixing	Biogas mixed with intermittent		Cattle manure	Increase in biogas production rate at 45.1%	[70]
	Hydraulic mixing	Slurry with intermittent		Cattle manure	Biogas production rate at 1.64 L/L	
		Sludge recirculation with intermittent		Food waste	Biogas production rate at 16.20 m^3^/d	[71]
Biochar addition	Manganese oxide-modified biochar (2.36 g/g VS)	Pyrolysis temperature at 600 °C for 120 min		Sludge dewatering	Increase in CH_4_ yield at 121.97	[72]
	Granular biochar (10 g/L)	Pyrolysis temperature at 800 °C for 8 h		Oil	Increase in CH_4_ yield at 32.5	[72]
	Fruitwood biochar	Pyrolysis temperature at 550 °C for 120 min		Chicken manure	Increase in CH_4_ yield at 69	[73]
	Corn stover biochar (0.25–1.0 g/day)	Pyrolysis temperature at 500 °C for 120 min		Primary sludge and waste-activated sludge	Increase in CH_4_ yield at 25	[74]
	Manure-derived biochar (10 g/L)	Pyrolysis temperature at 350 °C for 3 h		Dry dairy manure	Increase in CH_4_ yield at 24.9	[75]
	Citrus peel (0.5–1.5 g/g VS)	Pyrolysis temperature at 500 °C		Food waste and sludge	Increase in CH_4_ yield at about 89–151%	[76]

## Data Availability

No new data were created or analyzed in this study. Data sharing is not applicable to this article.
